# Predictors of burnout among resident doctors in a Nigerian teaching hospital

**DOI:** 10.4102/sajpsychiatry.v29i0.2017

**Published:** 2023-06-29

**Authors:** Mumeen O. Salihu, Alfred B. Makanjuola, Olatunji A. Abiodun, Amudalat T. Kuranga

**Affiliations:** 1 Department of Behavioural Sciences, University of Ilorin Teaching Hospital, Ilorin, Nigeria; 2 Department of Behavioural Sciences, Faculty of Clinical Sciences, University of Ilorin, Ilorin, Nigeria

**Keywords:** burnout, maslach burnout inventory, predictors, resident doctors, teaching hospital, Ilorin, Nigeria

## Abstract

**Background:**

Burnout is a psychological syndrome resulting from exposure to chronic work-related stress. There are, however, a few works of literature on burnout among trainee doctors in Nigeria.

**Aim:**

To determine the prevalence of burnout and its predictors among resident doctors across 16 medical specialties and/or subspecialties.

**Setting:**

The University of Ilorin Teaching Hospital (UITH), Ilorin, Nigeria.

**Methods:**

A cross-sectional study was conducted among 176 resident doctors between October 2020 and January 2021. The survey included the Proforma and Maslach Burnout Inventory-Human Services Survey for Medical Personnel (MBI-HSS MP).

**Results:**

The mean age of participants was 35.10 (SD 4.07) years. The prevalence of burnout was 21.6% for high emotional exhaustion (EE), 13.6% for high depersonalization (DP), and 30.7% for low personal accomplishment (PA). Being a younger resident doctor aged 31–35 (OR = 3.715, 95% CI [1.270 – 10.871]) was the only significant predictor for the EE. Predictors of DP included the age group 31–35 years (OR = 7.143, 95% CI [2.297 – 22.216]), duty hours >50 hours per week (OR = 2.984, 95% CI [1.203 – 7.401]), and presence of work-related stress (OR = 3.701, 95% CI [1.315 – 10.421]). A good relationship with colleagues negatively predicted low PA (OR = 0.221, 95% CI [0.086 – 0.572]).

**Conclusion:**

High levels of burnout are prevalent among resident doctors, comparable to international studies. Therefore, the government and other relevant stakeholders must drive legislation and formulate policies toward addressing the work-related factors associated with burnout in the Nigerian healthcare industry.

**Contribution:**

This study highlighted the determinants of burnout among Nigerian resident doctors, which necessitates targeted interventions to address them.

## Introduction

Burnout is a triad syndrome, made up of three components: emotional exhaustion (EE), depersonalisation (DP) and low personal accomplishment (PA), which usually results from chronic workplace stress.^1,2^ It may present with physical and psychological symptoms such as tiredness, headache, poor sleep, irritability, emotional instability and rigidity in relationships with other people.^3^ It may also be associated with depressive and/or anxiety symptoms, chronic pain syndromes or functional disorders of the cardiovascular or gastrointestinal system.^4,5^ Burnout has enormous negative consequences on doctors, patients and institutions.^6,7^ For example, physicians are more likely to abuse substances, become offensive or violent at work, suffer from depression and have higher suicide rates.^8,9^ For the institutions, there is low productivity because of increased staff turnover, absenteeism, poor performance and low motivation.^8^ The impact of burnout in medical personnel on patients includes decreased quality of medical care, decreased patient safety and increased medical errors.^10,11,12^

Resident doctors are early career doctors (ECDs) undergoing specialist training and combining academic and clinical work.^13,14,15^ Among the stages of a physician’s career, residency training is the peak time for burnout.^13,14^ This is because of high expectations of training, long working hours, prolonged sleep deprivation, uncontrolled schedules, high job demands and inadequate personal time often experienced by trainee doctors.^14,15,16,17^ The global prevalence of burnout among resident doctors is unclear because of different working conditions in various medical and surgical specialities.^6,16^

A meta-analysis of 47 eligible studies from North and South America (37), Europe (3), Asia (5), Australia (1) and Africa (1) showed an aggregate burnout prevalence of 51.0% among 22 778 medical and surgical residents with radiology (77.16%), neurology (71.93%) and general surgery (58.39%) being the top three specialties with the highest prevalence of burnout.^6^ Gouveia et al.^18^ in Brazil found 59.7%, 31.8% and 94.6% prevalence for high EE, high DP and low PA, respectively, among 129 medical residents. The independent predictors found were the presence of a stressful event within the previous 6 months and being a surgical resident.^18^ Abdulrahman and colleagues in UAE showed that 75.5% of resident doctors had moderate-to-high EE, 84% had high DP and 74% had a low PA.^19^ Also, Ashkar et al.^20^ in Lebanon reported that 80% of the residents had a high level of burnout in at least one domain of the Maslach Burnout Inventory (MBI), with 67.7% experiencing high EE. The predictors identified included being a female, long work hours (> 80 h per week), frequent call duty (> 8 call duties per month) and experiencing major stress.^20^

A systematic review of burnout studies comprising 412 Nigerian physicians between 1970 and 2017 found that only four articles met the inclusion criteria – the use of the Maslach Burnout Inventory-Human Services Survey (MBI-HSS) scale and being physicians.^7^ The aggregate prevalence of burnout was 23.6%–51.7%, with young age being a strong predictor.^7^ In Lagos, South-West, Nigeria, Ogundipe et al.^21^ found burnout rates of high EE (45.6%), high DP (57.8%) and low PA (61.8%) among 204 resident doctors. Emotional distress and a perceived heavy workload were the identified barriers that contributed to burnout.^21^ Ozumba and Alabere reported burnout rates of 23.8%, 14.7% and 30% as high EE, high DP and low PA, respectively, in a study involving 320 doctors and nurses in a tertiary hospital in Port Harcourt, South-South, Nigeria.^22^ The significant factors identified that contributed to burnout were salaries, professional grouping and days off in the work environment.^22^ The limitations of their study included the failure to compare burnout among specialties or departments, which may have given operational data for planning by the institution’s management.^22^ Furthermore, a study conducted among 220 primary care physicians in Jos, North-Central Nigeria, revealed 13.6%, 21.6% and 15% burnout rates as high EE, high DP and low PA, respectively.^23^ The predictors of burnout included being female, having an age range of 30–39 years, being a registrar or medical officer, working in a state specialist hospital and having poor perceived health status.^23^

In Nigeria, only limited studies on burnout among physicians have been conducted,^7,23^ with only a few among resident doctors in selected teaching hospitals.^7,21^ Also, there are fewer studies on burnout in the North-Central geo-political zone of the country (where the researchers practice) compared to the Southern regions^7^ and none among resident doctors in the University of Ilorin Teaching Hospital (UITH), Ilorin, Nigeria. The Ogundipe study on burnout among resident doctors in Lagos was a decade ago. The new healthcare reform laws like the *Medical Residency Training Act, 2017* (*MRTA 2017*) – the act that regulates the medical residency training programme in Nigeria, were adopted in the year 2018.^24^ Similarly, Nigeria’s healthcare industry has witnessed a mass exodus of resident doctors from their current training environment to seek employment abroad.^25^ This could further worsen the human resource capital with possible attendant burnout, medical errors and litigations. More so, there is an increasing rate of workplace violence against healthcare workers where resident doctors are physically attacked and injured by the relatives of patients.^26^ We, therefore, aimed to investigate burnout and its predictors among trainee doctors in UITH using standardised, validated MBI-HSS tool. The findings would add to the body of knowledge and provide information on possible stressors for burnout among this group of doctors for targeted intervention.

## Research methods and design

### Study design

A cross-sectional design study was used to gather data among resident doctors between October 2020 and January 2021.

### Study setting

The study was conducted in the 16 departments offering residency training in UITH, Ilorin, Nigeria. The specialties and/or departments were grouped further into Surgical (Anaesthesia, Otorhinolaryngology, Obstetrics and Gynaecology, Orthopaedic and Trauma, Ophthalmology, Radiology and Surgery), Medical (Epidemiology and Community Medicine, Family Medicine, Internal Medicine, Paediatrics and Psychiatry) and Laboratory subspecialties (Chemical Pathology, Haematology, Microbiology and Morbid Anatomy).

### Study population and sampling strategies

There were 245 resident doctors at the time of the study. The recruitment involved only respondents who gave informed consent and had been in the medical residency training (MRT) for at least 6 months. The 6 months minimum period of residency was to allow them to be familiar with the dynamics of the day-to-day activities of the training programme. Excluded were the resident doctors undergoing external rotation outside the hospital and those who were physically too ill to participate in the study. A total sampling of all consented eligible resident doctors was done. They were recruited through their respective departmental offices and grouped according to their training specialties for comparison. In each department, the doctors were listed and allocated numerical codes similar to that on the questionnaires. A total of 185 resident doctors were eligible, but only 176 participated in the study, with a response rate of 95.1%.

### Data collection

Respondents completed the Proforma and Maslach Burnout Inventory-Human Services Survey for Medical Personnel (MBI-HSS MP) questionnaires. The Proforma is a 47-item self-administered questionnaire designed by the authors, which included socio-demographic questions (age, gender, marital status, income, etc.), medical history, residency characteristics (specialty and year), workload-related factors (such as an average number of patients attended to per week, the average duration of call hours per week, duration of work hours per day, hours of sleep per week), recent stressors (work-related and non-work-related), harmful ideations (thoughts of death or thoughts of self-harm), job satisfaction, awareness of burnout phenomena and education or training in stress management.

The MBI-HSS MP is a 22-item self-administered questionnaire, generally considered the gold standard measure of burnout, developed by Maslach and Jackson.^27^ It comprises three subscales, namely: EE, DP and PA. The EE subscale has nine items measuring the reduced energy and job enthusiasm, emotional and cognitive distance from the job (e.g. ‘I feel emotionally drained by my work’), DP has five items measuring cynicism, lack of engagement and distancing from the patients, treatment of patients as inanimate objects (e.g. ‘I feel I look after certain patients or clients impersonally as if they are objects’).^27^ Personal accomplishment has eight items measuring the perception of influencing others, working well with others and dealing well with problems (e.g. ‘I have accomplished many worthwhile things in this job’).^27^ The response scales range from a score of zero (never) to six (every day). The respondents indicated the frequency with which they experience certain feelings related to their work on the 7-point fully anchored scale.

The MBI inventory measures levels of burnout as either high, moderate or low for each of the three domains. The direct scoring method is used for the items of the EE and DP by adding the values of the ratings shaded on the frequency domain, while reverse scoring is used for the items of the PA domain by adding the reverse values of the shaded ratings. The level of burnout is high if EE is ≥ 27, DP is ≥ 13 and PA is ≤ 31; moderate if EE is 17–26, DP is 7–12 and PA is 32–38 and low if EE is ≤ 16, DP is ≤ 6 and PA is ≥ 39. The psychometric properties of the MBI have been established in Nigeria with Cronbach’s alpha of 0.86 and concurrent validity coefficients in the range of 0.01–0.36.^28^

### Data analysis

All analyses were performed using Statistical Package for Social Sciences version 20 (SPSS-20). Frequency tables and charts were used to report descriptive statistics and cross-tabulations to show relationships between variables. Chi-square was used to compare associations between the domains of burnout and independent variables, with Yates’ Correction and Bonferroni’s correction for multiple testing (*p*-value < 0.01). Multivariate logistic regression was used to determine predictive variables. A *p*-value of ≤ 0.05 was used in selecting the variables for logistic regression analysis.

### Ethical considerations

The study was approved by the UITH Ethics and Research Committee (ERC) (Approval No: ERC PAN/2020/06/0023).

## Results

### Socio-demographic characteristics of the respondents

There were more male resident doctors than females (69.9% vs. 30.1%) with a mean age of 35.10 ± 4.07 (range: 26–48 years). Most (85.2%) were married, and many (54.2%) had 1–2 children. About two-thirds (65.9%) of residents were unsatisfied with their income.

### Residency and work-related characteristics

Participation rates among respondents differed across specialties, with surgical specialties being the highest (*n* = 85; 48.3%), followed by medical-related disciplines (*n* = 81; 46.0%) and laboratory departments (*n* = 10; 5.7%). The senior registrars were more than half (55.1%) of the total participants and 30.7% of residents were in their 2nd year of residency training. The average weekly mean duration of work hours was 55.21 ± 28.06. More than half (52.8%) of the respondents reported poor sleep, with a mean average number of 2.92 ± 1.53 days of restorative sleep in a week. Good sleep in this study refers to the perceived feeling of restorative sleep for at least four days a week, while poor sleep is a perceived restorative sleep for fewer than 4 days a week.

The majority (75.6%) perceived their job environment as stressful. Also, 82.4% of the respondents experienced stressful events within the previous 6 months from the time of the survey. Work-related stress was reported by 54% of the respondents. Most (88.1%) of residents were satisfied with their relationship with their colleagues. However, most participants (79.5%) were dissatisfied with their training programme. Nearly all (95.5%) residents had received no stress management training during their residency training in the hospital, and 98.3% voiced that they wanted hospital management to organise stress management training in the hospital.

### Prevalence of burnout among resident doctors

**FIGURE 1:**
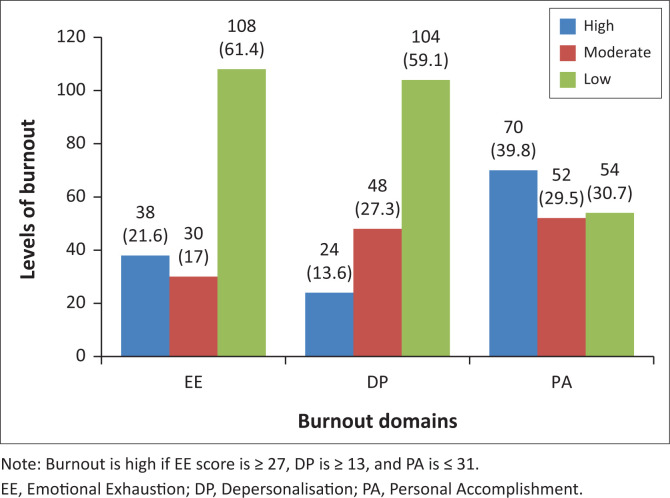
Burnout levels using Maslach Burnout Inventory-Human Services Survey for Medical Personnel.

Thirty-eight (21.6%) of the residents had high EE; 24 (13.6%) showed high DP, and 54 (30.7%) reported low PA. Overall, the prevalence of burnout syndrome was 30.7% in one domain only, 10.2% in at least two domains and 2.3% when all three domains were considered (Figure 1).

### Socio-demographic variables associated with burnout among resident doctors

#### Association between socio-demographic factors and emotional exhaustion of burnout

There was no significant association between burnout in the domain of high EE and socio-demographic variables. High EE was higher among younger resident doctors aged ≤ 30 years than those ≥ 41 years (χ^2^ = 12.652, *p* = 0.049). However, this was not statistically significant when Bonferroni correction was applied (*p* ≤ 0.01).

#### Association between socio-demographic factors and depersonalisation of burnout

The DP domain of burnout was significantly higher in respondents ≤ 30 years of age (χ^2^ = 23.84, *p* = 0.001). Other socio-demographic variables were not associated with DP.

#### Association between socio-demographic factors and personal accomplishment of burnout

There was no significant association between low PA and socio-demographic factors. Male residents had a higher sense of PA than females (45.5% vs. 26.4%; *p* = 0.028). However, this was insignificant when the Bonferroni correction was applied (*p* ≤ 0.01).

#### Association between work-related factors and burnout

**Association between work-related factors and emotional exhaustion of burnout:** More participants who reported poor sleep had significantly higher EE than those who had a good sleep (χ^2^ = 19.302, *p* = < 0.001). Similarly, the presence of any stressful events within the previous 6 months (χ^2^ = 7.496; *p* = 0.006) and work-related stress (χ^2^ = 7.160; *p* = 0.007) had significant associations with the EE domain of burnout. There was no significant inter-departmental difference in the EE domain (Table 1).

**TABLE 1:** Relationships between work-related factors and emotional exhaustion of burnout.

Variables	Emotional exhaustion burnout						*χ* ^2^	*P*
	High (38)		Moderate (30)		Low (108)			
	*n*	%	*n*	%	*n*	%		
**Rank**								
Registrar	22	27.8	15	19.0	42	53.2	4.487	0.106
Senior registrar	16	16.5	15	15.5	66	68.0	-	-
**Duration of calls per week (hours)**								
< 20	3	10.0	7	23.3	20	66.7	6.294	0.614
20–39	10	27.0	3	8.1	24	64.9	-	-
40–59	14	20.6	12	17.6	42	61.8	-	-
60–79	8	27.6	6	20.7	15	51.7	-	-
≥ 80	3	25.0	2	16.7	7	58.3	-	-
**Duration of work per week (hours)**								
≤ 50	18	18.2	16	16.2	65	65.7	2.001	0.368
> 50 h	20	26.00	14	18.2	43	55.8	-	-
**Break during working hours**								
Yes	4	11.8	11	32.4	19	55.8	7.884	0.019
No	34	23.9	19	13.4	89	62.7	-	-
**Departments**								
Laboratories	2	20.0	1	10.0	7	70.0	2.470	0.650
Medicals	14	17.3	16	19.8	51	63.0	-	-
Surgical	22	25.9	13	15.3	50	58.8	-	-
**Sleep well**								
Yes	9	10.8	9	10.8	65	78.4	19.302	< 0.001
No	29	31.2	21	22.6	43	46.2	-	-

*p* value < 0.01 is statistically significant (Bonferroni correction was applied).

*χ*^2^, Chi square test.

**Association between work-related factors and depersonalisation of burnout:** High DP was significantly observed among resident doctors working more than 50 h per week (χ^2^ = 11.546; *p* = 0.003). Also, respondents with poor sleep (χ^2^ = 18.376; *p* = < 0.001) and those experiencing work-related stress (χ^2^ = 6.795; *p* = 0.009) reported significantly higher DP. There was no significant difference in residency cadre and specialities with the DP domain of burnout (Table 2).

**TABLE 2:** Relationships between work-related factors and depersonalisation of burnout.

Variables	Depersonalisation				*χ* ^2^	*P*
	High (24)		Moderate to low (152)			
	*n*	%	*n*	%		
**Stressful job environment**						
Yes	23	17.3	110	82.7	6.181	0.013
No	1	2.3	42	97.7	-	-
**Presence of stressful events in the last 6 months**						
Yes	23	15.9	122	84.1	3.463	0.063
No	1	3.2	30	96.8	-	-
**Work related stress**						
Yes	19	19.8	77	80.2	6.795	0.009
No	5	6.3	75	93.8	-	-
**Academic related stress**						
Yes	11	21.2	41	78.8	3.542	0.060
No	13	10.5	111	89.5	-	-
**Attempts at exams**						
Yes	9	8.7	94	91.3	5.060	0.024
No	15	20.5	58	79.5	-	-
**Satisfaction with relation with colleagues**						
Yes	21	13.5	134	86.5	0.009	0.926
No	3	14.3	18	85.7	-	-
**Satisfaction with training programme**						
Yes	2	5.6	34	94.4	2.509	0.113
No	22	15.7	118	84.3	-	-
**Frequency of medical errors**						
Sometimes	8	16.7	40	83.3	1.579	0.454
Rarely	13	11.4	101	88.6	-	-
Never	3	21.4	11	78.6	-	-
**Job satisfaction**						
High	2	12.5	14	87.5	4.234	0.120
Medium	6	7.9	70	92.1	-	-
Low	16	19.0	68	81.0	-	-

*p* < 0.01 is statistically significant (Bonferroni correction was applied).

*χ*^2^, Chi square test.

**Association between work-related factors and personal accomplishment of burnout:** The majority (61.9%) of respondents who were not satisfied with their relationship with colleagues had significantly low PA compared to 26.5% of those who reported having satisfactory relationships with colleagues (χ^2^ = 10.930; *p* = 0.001). Similarly, 34 (40.5%) participants with low job satisfaction had low PA compared to 19 (25.0%) and 1 (6.3%) with medium and high job satisfaction, respectively (χ^2^ = 9.433; *p* = 0.009). However, the residency cadre and specialties did not show an association with PA (Table 3).

**TABLE 3:** Relationships between work-related factors and personal accomplishment of burnout.

Variables	Personal Accomplishment Burnout						*χ* ^2^	*P*
	Reduced (54)		Moderate (52)		High (70)			
	*n*	%	*n*	%	*n*	%		
**Rank**								
Registrar	24	30.4	22	27.8	33	41.8	0.288	0.866
Senior registrar	30	30.9	30	30.9	37	38.2	-	-
**Duration of calls per week (hours)**								
< 20	9	30.0	6	20.0	15	50.0	8.653	0.372
20–39	13	35.1	16	43.2	8	21.6	-	-
40–59	18	26.5	20	29.4	30	44.1	-	-
60–79	10	34.5	7	24.1	12	41.4	-	-
≥ 80	4	33.3	3	25.0	5	41.7	-	-
**Duration of work per week (hours)**								
≤ 50	31	31.3	29	29.3	39	39.4	0.042	0.979
> 50	23	29.9	23	29.9	31	40.2	-	-
**Break during working hours**								
Yes	12	35.3	9	26.5	13	38.2	0.452	0.798
No	42	29.6	43	30.3	57	40.1	-	-
**Departments**								
Laboratories	4	40.0	4	40.0	2	20.0	1.802	0.772
Medicals	24	29.6	23	28.4	34	42.0	-	-
Surgical	26	30.6	25	29.4	34	40.0	-	-
**Sleep well**								
Yes	27	32.5	21	25.3	35	42.2	1.359	0.507
No	27	29.0	31	33.3	35	37.6	-	-

*p* value < 0.01 is statistically significant (Bonferroni correction was applied).

*χ*^2^, Chi square test.

## Predictors of burnout among respondents

### Predictors of emotional exhaustion domain of burnout

Table 4 indicates that the age group (31–35 years) of respondents was the only predictor of high EE (OR = 3.715, 95% CI [1.270–10.871]).

**TABLE 4:** Predictors of emotional exhaustion: Multivariate logistic regression analysis.

Variables	*β*	Odds ratio	95% CI
**Age groups (years)**			
≤ 30	RC	-	-
31–35	1.312	3.715	1.270–10.871
36–40	0.921	2.512	0.889–7.097
≥ 41	2.012	7.475	0.795–70.302
**Break during work hours**			
No	RC	-	-
Yes	−0.859	0.424	0.139–1.288
**Sleep well**			
Yes	RC	-	-
No	−1.315	0.288	0.118–0.609
**Stressful job environment**			
Yes	−1.030	0.357	0.112–1.134
No	RC	-	-
**Presence of stressful events in the last 6 months**			
Yes	2.033	7.636	0.095–64.422
No	RC	-	-
**Work related stress**			
Yes	−0.347	0.707	0.291–1.720
No	RC	-	-
**Attempts at exams**			
Yes	−0.714	0.489	0.237–1.012
No	RC	-	-

CI, Confidence Interval; RC, Reference Category; *β*, Coefficient of logistic regression.

### Predictors of depersonalisation domain of burnout

Participants within the age range of 31–35 years had increased odds of experiencing burnout in the domain of DP than those ≤ 30 years (OR = 7.143, 95% CI [2.297–22.216]). Similarly, duty hours > 50 h per week (OR = 2.984, 95% CI [1.203–7.401]) and work-related stress (OR = 3.701, 95% CI [1.315–10.421]) were predictive of high DP (Table 5).

**TABLE 5:** Predictors of depersonalisation: Multivariate logistic regression analysis.

Variables	*β*	Odds ratio	95 % CI
**Age groups (years)**			
≤ 30	RC	-	-
31–35	1.966	7.143	2.297–22.216
36–40	1.935	6.923	2.224–21.550
≥ 41	20.941	1.242	0.067–4.973
**Marital status**			
Single	RC	-	-
Married living together	0.886	2.424	0.764–7.697
Married living apart	−0.570	0.565	0.158–2.030
**Duration of work per week (hours)**			
≤ 50	RC	-	-
> 50	−1.093	2.984	1.203–7.401
**Sleep well**			
Yes	RC	-	-
No	−2.530	0.080	0.018–0.351
**Stressful job environment**			
Yes	−1.733	0.177	0.023–1.362
No	RC	-	-
**Work related stress**			
Yes	1.309	3.701	1.315–10.421
No	RC	-	-
**Attempts at exams**			
Yes	−0.994	0.370	0.152–0.900
No	RC	-	-

CI, Confidence Interval; RC, Reference Category; *β*, Coefficient of logistic regression.

### Predictors of personal accomplishment domain of burnout

Table 6 showed that only participants that reported having satisfactory relationships with their colleagues were less likely to have low PA (OR = 0.221, 95% CI [0.086–0.572]).

**TABLE 6:** Predictors of reduced personal accomplishments: Multivariate logistic regression analysis.

Variables	*β*	Odds ratio	95 % CI
**Gender**			
Female	−0.093	0.911	0.455–1.823
Male	RC	-	-
**Satisfaction with colleagues**			
Yes	−1.508	0.221	0.086–0.572
No	RC	-	-
**Job satisfaction**			
High-Moderate	RC	-	-
Low	−0.448	0.639	0.460–0.889

CI, Confidence Interval; RC, Reference Category; *β*, Coefficient of logistic regression.

## Discussion

This study investigated the prevalence of burnout, associated factors and independent predictors among resident doctors in UITH, Ilorin, Nigeria.

### Prevalence of burnout among resident doctors

The prevalence of burnout found in this study was 30.7%, 10.2% and 2.3% in at least one domain, two domains and three domains, respectively. The overall prevalence of burnout was 2.3% among doctors that participated. The rate is lower than the 4.7% reported by Ozumba and Alabere in Nigeria.^22^ The difference observed may be because of the large sample size (N = 320) and the inclusion of nurses in the latter study. However, the burnout rate of 2.3% is much lower than the 11.7%, 12.2%, 13.1% and 27.9% reported among resident doctors in Yemen,^29^ Pakistan,^30^ Australia^31^ and Brazil,^18^ respectively. The differences may be because of the low sample size (N = 82) in the study by Zubairi and Noordin in Pakistan. Also, the threshold criteria used for burnout were lower in the studies by Leung et al.^31^ in Australia and Al-Dubai et al.^29^ in Yemen compared to the present study. Our study found a prevalence of 30.7% in at least one domain, consistent with local findings.^22,23^ However, the rate is lower than that reported in Pakistan (74.4%),^30^ South Africa (76%),^32^ Beirut (80%)^20^ and Syria (93.75%).^33^

With regard to the level of burnout, 21.6% of residents had high EE, 13.6% experienced high DP and 30.7% reported low PA. This is in keeping with findings from one local study.^22^ Dunwoodie and Auret in Australia reported that 22.5%, 7.5% and 2.5% of respondents experienced high EE, high DP and low PA, respectively.^34^ This finding is fairly comparable to the present study, except in the PA domain.^34^ This would suggest that doctors in Australia have a higher sense of PA than those in UITH Ilorin. The Australian study involved a single specialty (palliative care medicine) with small sample size. One study also opined that there are many rewards in palliative medicine practices, like the relationships achieved with patients and feelings of competence in symptom control and managing death and dying well, which may be protective and could explain this difference.^35^ Nonetheless, a study conducted among Canadian radiology residents found that 35.9% of the respondents had low PA, which is comparable to 30.7% in the present study.^36^ In contrast, Ogundipe et al.^21^ reported a prevalence of 45.6%, 57.8% and 61.8% in the domains of high EE, high DP and low PA, respectively, higher than the findings in the present study. In the USA, 44.4% of resident doctors had high EE, 50.7% had high DP and 22.0% had a low PA.^13^ The abridged MBI scale used in the USA study could have contributed to the rate difference. Also, the present study was conducted about 8 months after the reported first case of coronavirus disease 2019 (COVID-19) in Nigeria when core clinical activities were vastly scaled-down and the introduction of virtual meetings and training as part of the *new normal* to sustain learning and minimise the community spread of severe acute respiratory syndrome coronavirus 2 (SARS-COV-2) infection in training institutions in line with the national COVID-19 protocol for Nigeria.^37^ These undoubtedly temporarily reduced the workload significantly and slowed traditional training activities down, which could have affected the findings in the current study when compared to the study by Ogundipe et al.^21^

### Socio-demographic factors associated with burnout among respondents

Being a resident doctor aged ≤ 30 years was the only significant variable associated with burnout in the domain of DP among the socio-demographic factors. Some authors posited that younger residents usually have lower stress-coping capacities and may be less experienced, which predisposes them to high burnout in the area of EE and DP when compared to older residents.^4,38,39^ Ogundipe and colleagues reported a similar association in the area of low PA.^21^ However, Stanetić and Tesanović reported that older age (> 46 years) was a stronger predictor of a high EE domain of burnout among physicians.^40^ Older residents are more likely to be married and could struggle with family and residency commitments, resulting in work–life imbalance and burnout.^41^ However, other authors did not find an association between burnout and age.^18,22^

This study found that being a male resident doctor was associated less with low PA than females. The difference was not significant following the Bonferroni correction and was consistent with previous studies.^21,22,42^ However, Low et al.^6^ in Singapore and Pindar et al.^43^ in Nigeria showed that being male was a significant moderator of burnout, with a significant relationship in the domain of low PA. Similarly, Alhaffar et al.^33^ in Syria reported that being a male resident doctor strongly predicted burnout in the EE domain. A meta-analysis of the results showed that women reported burnout in the domain of EE, while men experienced burnout in the DP domain.^44^

The study also found no association between marital status and burnout, which is in keeping with previous studies.^18,21^ However, Chen et al.^38^ found that being a married physician was significantly associated with burnout in the EE domain. One study revealed that marriage and family provided solid social support and better-coping skills for physicians, thereby limiting exposure to burnout.^4^

### Work-related factors associated with burnout among respondents

The study indicates that poor sleep and work-related stress were significantly associated with burnout in both domains of EE and DP. The presence of a stressful event within the previous 6 months and working hours (> 50 h per week) were significantly related to high EE and high DP, respectively. Similarly, poor satisfaction with relationships with colleagues and low job satisfaction were significantly related to the low PA domain of burnout.

Long working hours are essential to postgraduate residency training as it allows total exposure to a broad range of cases and clinical scenario and helps develop proficiency in independent decision-making.^45^ Also, working hours have been at the centre of the burnout epidemic as respondents who worked for > 50 h per week had significantly high DP in our study. This finding is similar to the study by Tung et al.,^46^ who found that physicians with > 60 work h per week had a significantly high level of burnout. Similarly, Chen et al.^38^ reported that physicians with long continuous working hours experienced high levels of DP. The authors found that physicians who worked for 13 h–17 h continuously experienced high levels of EE.^38^ However, Zubairi and Noordin did not show a significant association between the actual length of work hours and burnout.^30^ In the same vein, Ripp et al.^47^ in a comparative study did not show improvement in job burnout following the enforcement of limited work hours regulation for internal medicine residents from three different training institutions in the USA. Nonetheless, Barden et al.^10^ found that the most surgical residents significantly improved their quality of life, with significant improvement in American Board of Surgery In-Training Examination (ABSITE) scores for junior residents compared to senior residents after the implementation of duty hours restriction regulation.

Respondents who suffered a stressful event within the previous 6 months had a highly significant EE than those who did not report any last stressful event. This is in agreement with the findings by Gouveia et al.^18^ in Brazil and Martini et al.^48^ in the USA. Previous studies^31,38,49^ have reported a significant association between low job satisfaction and low PA, which is consistent with the findings in the present study. The present study also found a significant association between relationship satisfaction among colleagues and low PA, in keeping with results from previous researchers.^21,30^

### Predictors of burnout among resident doctors in the study location

The present study showed that respondents’ age, duty hours > 50 h per week, work-related stress and satisfaction with colleague relationships were independent predictors of burnout. Specifically, respondents aged 31–35 compared to those ≤ 30 years had a 3.7-fold and 7.1-fold risk of developing high EE and DP, respectively. Campbell and colleagues found significant burnout among young American surgeons.^8^ This is because younger surgeons might be experiencing more emotional unrest because of social expectations regarding work-life balance, personal growth and development.^8^ A Yemen study found that those aged ≤ 29 years had a 4.1-fold risk of demonstrating a high level of burnout than those aged ≥ 40 years.^29^

The study showed that participants with > 50 h of duty weekly had about three times the chance of experiencing burnout in the DP subscale than those with ≤ 50 h per week. This is in agreement with the study by Chen et al.^38^ in Taiwan, where physicians that worked for ≥ 65 h per week had a 1.4-fold risk of low-level burnout compared to those who worked 49 h–56 h per week and a 1.5-fold risk of moderate-level burnout in EE domain. Also, Al-Dubai et al.^29^ reported that those who worked ≤ 40 h per week had two times the odds of experiencing burnout compared to those with > 40 h of weekly duty. Martini and colleagues found a significant association between burnout and duty > 80 h per week.^50^ However, some studies found no relationship between work hours and burnout.^30,48^

Furthermore, interpersonal and professional conflicts are significantly associated with burnout, as shown in the present study. Lasebikan and Oyetunde found that conflict between doctor and/or nurse increases the odds of experiencing burnout in each of EE and DP domains by three-fold.^51^ Embriaco and colleagues also showed that perceived conflicts and poor co-worker relationships were independent risk factors for severe burnout.^3^ A strong commitment to preventing conflicts and improving communication among hospital staff by relevant stakeholders may be imperative to reduce the risk of burnout and enhance optimum patient care.

### Strengths and limitations

The study used a sufficient sample size (95.1% response rate) and standardised, validated tools, expected to have strengthened the findings. The study’s limitation is the cross-sectional design, which makes establishing the temporality and causality of the observed relationships difficult. Recall and response bias were also applicable because of self-reported questionnaires where some participants might respond in socially desirable ways, leading to possible underreporting of burnout. It is a single-centre study in one out of six geo-political zones in Nigeria. It thus can only be generalised to some of the resident doctors in training in Nigeria. Therefore, it suggests a more extensive multicentre study covering the country’s six geo-political zones. The study took place at the peak of COVID-19 restrictions, during which workload was much reduced for doctors in Ilorin. This might suggest that the prevalence for burnout in the study population might actually be higher than what was obtained. Also, future studies can look at the objective assessment of sleep problems among trainee doctors using validated tools.

## Conclusion and recommendations

This study revealed a high prevalence rate of burnout (30.7%) in at least one domain of MBI among resident doctors in UITH, Ilorin, Nigeria. The predictors of burnout identified included being a resident doctor aged 31–35 years, duty hours > 50 h per week, work-related stress and poor satisfaction with relationships with colleagues. The findings underscored the role of organisational stressors and provided a better understanding of the context in which burnout occurs, which could allow for appropriate interventions. The researchers, therefore, suggest an urgent need for the practices of mental health friendly policies at the workplace with an emphasis on the prevention of burnout for better quality service delivery. There is a need to enforce a limit to the maximum unit of call hours for resident doctors. The promotion of good interpersonal relationships and professional harmony should be encouraged. Further work would include the impact of culture and traditional gender roles on the association between gender and low PA. Lastly, a comparative study is warranted to assess the quality of training received in the new methods (virtual meetings and training) versus traditional training methods. If found similar, the new training methods should be adapted, which could lead to less burnout.
